# Identification of Lesional Tissues and Nonlesional Tissues in Early Gastric Cancer Endoscopic Submucosal Dissection Specimens Using a Fiber Optic Raman System

**DOI:** 10.1155/2020/8015024

**Published:** 2020-05-15

**Authors:** Zhaohui Luan, Yusi Qin, Jianhua Dai, Hongbo Wu, Yao Chen, Xiaofeng Feng, Guiyong Peng

**Affiliations:** Endoscopic Center, Department of Gastroenterology, Third Military Medical University, Southwest Hospital, China

## Abstract

**Aim:**

To identify lesional and nonlesional tissues from early gastric cancer (EGC) patients by Raman spectroscopy to build a diagnostic model and effectively diagnose EGC.

**Method:**

Specimens were collected by endoscopic submucosal dissection from 13 patients with EGC, and 55 sets of standard Raman spectral data (each integrated 10 times) were obtained using the fiber optic Raman system; there were 33 sets of lesional tissue data, including 18 sets of high-grade intraepithelial neoplasia (HGIN) data and 15 sets of adenocarcinoma data, and 22 sets of nonlesional tissue data. After the preprocessing steps, the average Raman spectrum was obtained.

**Results:**

The nonlesional tissues showed peaks at 891 cm^−1^, 1103 cm^−1^, 1417 cm^−1^, 1206 cm^−1^, 1234 cm^−1^, 1479 cm^−1^, 1560 cm^−1^, and 1678 cm^−1^. Compared with the peaks corresponding to nonlesional tissues, the peaks of the lesional tissues shifted by different magnitudes, and a new characteristic peak at 1324 cm^−1^ was observed. Comparing the peak intensity ratio and the integral energy ratio of the lesional tissues with those of the nonlesional tissues revealed a significant difference between the two groups (independent-samples*t*-test, *P* < 0.05). Considering the peak intensity ratio of I1560 cm^−1^/I1103 cm^−1^ as a diagnostic indicator, the accuracy, sensitivity, and specificity of diagnosing EGC were 98.8%, 93.9%, and 91.9%, respectively. Considering the integral energy ratio (noncontinuous frequency band and continuous frequency band) as a diagnostic indicator, the accuracy, sensitivity, and specificity of diagnosing EGC were 99.2-99.6%, 93.9-97.0%, and 95.5%, respectively.

**Conclusions:**

The integral energy ratio of the Raman spectrum could be considered an effective indicator for the diagnosis of EGC.

## 1. Introduction

Gastric cancer is one of the most common malignancies in the world, and its incidence and mortality rates continue to be high, making it a serious threat to human health. According to the latest statistics, there were approximately 1,033,000 new gastric cancer cases and 783,000 related deaths in the world in 2018, ranking gastric cancer sixth in incidence and third in mortality among malignancies [1]. China is a country with a high incidence of gastric cancer, accounting for approximately 50% of all incident cases of gastric cancer and cases of related mortality globally. The 5-year survival rate of gastric cancer is low, at approximately 25%-30%, but the 5-year survival rate of early gastric cancer (EGC) is 90%, much higher than the rate of 15% for advanced gastric cancer [2–4]. Therefore, the early detection, early diagnosis, and early treatment of gastric cancer are essential.

Currently, the methods for diagnosing gastric cancer mainly include serology, imaging, and endoscopic biopsy. Among these methods, the specificity and sensitivity of serological tests are poor, and increases in indexes mostly appear in advanced gastric cancer, causing this approach to be used more often as an auxiliary indicator for diagnosing advanced gastric cancer and monitoring for recurrence and progression after gastric cancer surgery in clinical work [5]. Imaging examinations are expensive, making them more valuable in determining tumor size, eliminating the invasion of adjacent structures, evaluating metastatic tumors, and assessing tumor biology and response than in diagnosing EGC [5], for which imaging is poorly suited. Compared to the other approaches, endoscopy, currently the main method for diagnosing EGC, including common endoscopy plus biopsy, magnifying endoscopy (ME), narrow-band imaging endoscopy (NBI), and magnifying endoscopy with narrow-band imaging (ME-NBI), is more dependent on the doctor's experience and subjective judgment. Endoscopic biopsy is the gold standard for clinical diagnosis, but the invasiveness of the operation, the long waiting time, the difficulty in collecting lesional tissue, and some other shortcomings still exist. Additionally, the lack of obvious characteristics of lesions under endoscopy in EGC increases the risk of missed diagnosis.

Raman spectroscopy is an inelastic scattering technique that is performed by shifting the frequency of material molecular vibrations with incident light. The obtained Raman spectra exhibit different characteristics as the molecular content and composition of the material change. The structure, content, and conformation of various components in cells will change to different extents during the process of carcinogenesis; thus, Raman spectroscopy could be used to identify differences between the spectra obtained from early cancer tissues and those obtained from healthy tissues [6, 7]. Therefore, real-time Raman detection could be a rapid, noninvasive, objective, simple, accurate, and specific detection method.

Raman spectroscopy has been widely used in the diagnosis of various tumors, such as skin tumors and oral tumors and tumors of gastric cancer, esophageal cancer, ovarian cancer, and prostate cancer [8–13]. Most articles report that biopsy specimens are used for testing, while large tissues are rarely measured. Here, we performed endoscopic submucosal dissection (ESD) in patients with EGC and acquired Raman spectra from the specimens after surgery to explore the feasibility of using fiber optic Raman spectroscopy to distinguish EGC tissues from nonlesional tissues among ESD specimens.

## 2. Materials and Methods

### 2.1. Fiber Optic Raman Spectroscopy System

**Figure 1:**
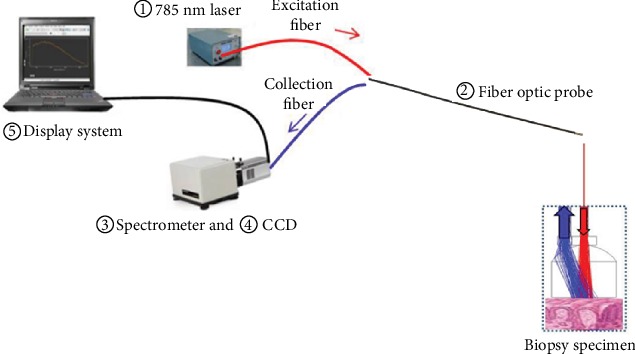
Fiber optic Raman spectroscopy system.

(1) A near-infrared semiconductor laser with an excitation wavelength of 785 nm (Changchun New Industries Optoelectronics Tech, China). (2) A fiber optic Raman probe (Em Vision, America): the Raman fiber optic bundle was 3 m in length and had an outer nylon protective sleeve composed of 7 collection fibers (300 *μ*m in diameter, N.A. = 0.22) surrounding the central excitation fiber (272 *μ*m in diameter, N.A. = 0.22), with two filters incorporated at the proximal and distal ends of the probe to maximize the collection of tissue Raman signals while reducing interference from light other than Raman-scattered light. (3) A Raman imaging spectrometer (Andor, England). (4) A charge-coupled device (CCD) camera (Andor, England). (5) A monitor (Lenovo, China). (6) Andor SOLIS software. The fiber optic Raman spectroscopy system is shown in Figure 1.

### 2.2. Function of the System

Usually, a 785 nm near-infrared laser beam passes through the excitation fiber and vertically irradiates the surface of the specimen. The scattered light is collected by the surrounding collection fibers and transmitted back to generate an optical image by the Raman imaging spectrometer. After the optical signal is converted into a recognizable electrical signal in the CCD camera, a visual Raman image is displayed on the screen of the monitor. Each Raman spectrum could be obtained in 1 second. Each site was probed 10 times to yield an average integrated Raman spectrum.

**Figure 2:**
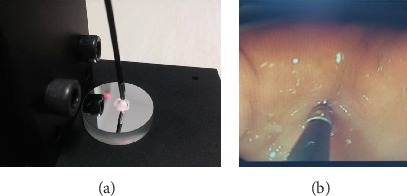
Representative images of the fiber optic Raman system being used (a) in vitro and (b) in vivo.

According to requirements, the fiber optic Raman spectroscopy system can be applied both in vitro and in vivo. When applied in vitro, the fiber optic Raman probe is vertically pressed against the surface of the tissue sample in a dark environment at a normal temperature to obtain Raman spectral data. When applied in vivo, the fiber optic Raman probe is placed against the surface of the tissue to be measured inside the body through the endoscopic biopsy hole, and Raman spectral data are obtained after maintaining the stability of the endoscope lens and the fiber optic probe for 10 seconds. Representative images of the fiber optic Raman system being used in vitro and in vivo are shown in Figure 2.

### 2.3. Sampling

ESD specimens were collected from 13 patients after EGC surgery at the endoscopic center of the Third Military Medical University Southwest Hospital. The histopathological results indicated 6 cases of high-grade intraepithelial neoplasia (HGIN) and 7 cases of adenocarcinoma. All patients in the study volunteered and signed informed consent forms permitting the investigative collection of ESD specimens and Raman spectral data. The study was performed in accordance with the human trial ethics standards and was approved by the hospital ethics committee.

**Figure 3:**
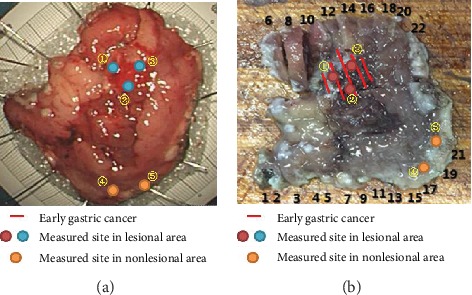
An EGC ESD specimen: (a) fresh specimen without any processing; (b) specimen with its shape restored after histopathological sectioning, along with the measured sites (①–⑤).

**Figure 4:**
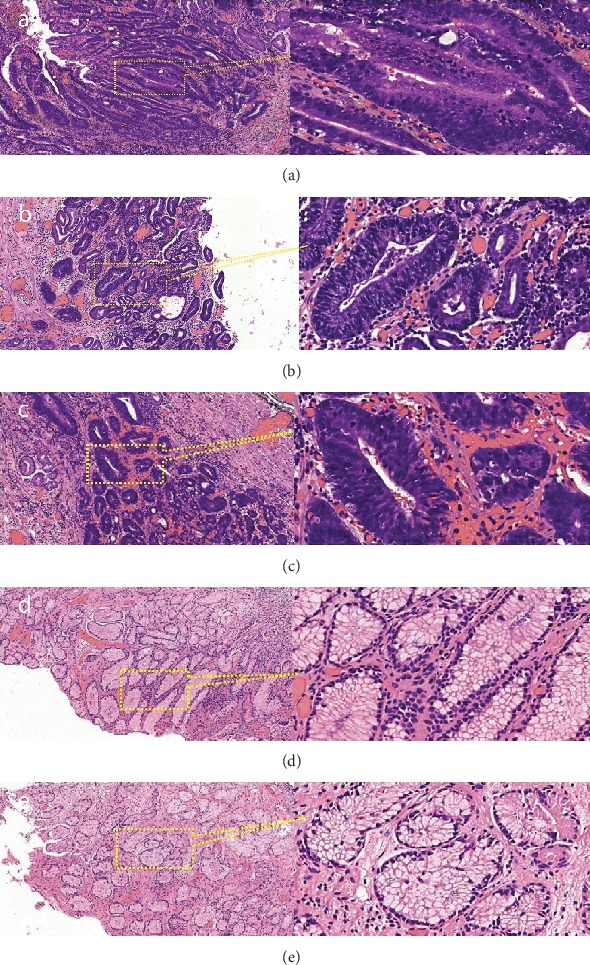
Histopathological results for different measured sites in ESD specimens: (a–c) measured sites in adenocarcinoma tissue; (d, e) measured sites in nonlesional tissue.

ESD specimens were immediately sent to the Raman spectrum collection room without any processing after using ME to determine the most severely affected area of the lesional tissue. Then, the Raman spectral range was set as 0-2000 cm^−1^, the exposure time was set as 1 second, the number of integrations was set as 10, the CCD temperature was set as -80°C, and the electrical current was set as 66 mA. The specimen was placed on an aluminized mirror, and then, the most severely affected area of the lesional tissue was selected after observation by ME. Next, 2-5 points were selected depending on the size of the most severely affected area. After the measurement was completed, the site of the measured point was marked accurately. Then, several sites of nonlesional tissue around the lesional tissue were selected and measured by the same method. After each measurement was completed, the site of the measured point was marked accurately. The specimen was fixed, soaked in 10% formalin solution, and sent to a histopathologist for double-blind histopathological examination. According to the histopathological results, we restored the shape of the ESD specimen, determined the area confirmed as EGC and excluded data that did not meet the criteria (standard: the site of the measured point we marked located in the lesional area was confirmed as EGC by the histopathologist; the site of the measured point we marked located in the nonlesional area was confirmed as normal tissue by the histopathologist). A total of 55 sets of Raman spectral data were collected, including 15 sets of HGIN Raman spectral data, 18 sets of adenocarcinoma Raman spectral data, and 22 cases of nonlesional Raman spectral data.  Figure 3 shows an example of an EGC ESD specimen.  Figure 4 shows the histopathological results for different measured sites.

### 2.4. Spectral Data Processing

**Figure 5:**
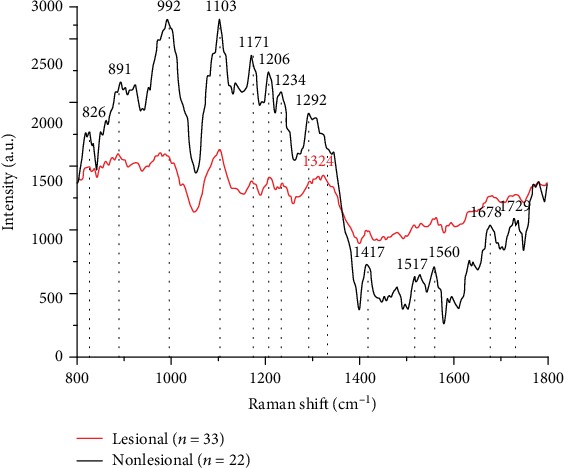
Mean Raman spectra of 33 EGC tissue specimens (red line) and 22 nonlesional tissue specimens (black line).

**Figure 6:**
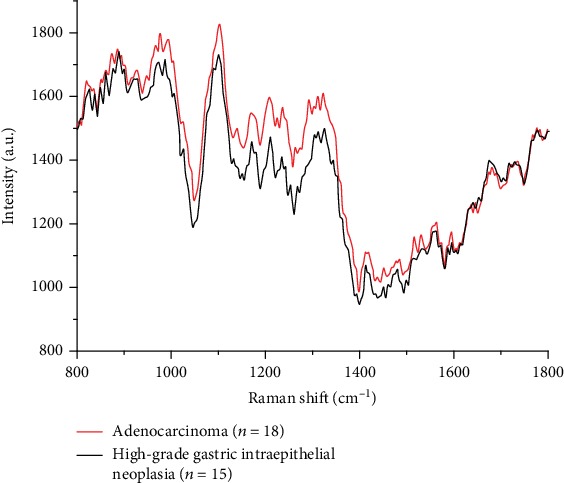
Mean Raman spectra of 18 adenocarcinoma tissue specimens (red line) and 15 HGIN tissue specimens (black line).

Due to the background autofluorescence of tissue and external disturbances, the original measured Raman spectral data were at a high baseline level, and the Raman spectral signal was weak. The data format was converted by Andor SOLIS, the baseline was lowered, and the strong background fluorescence was removed by Origin Pro 8.0 software. Next, the Raman signal was highlighted, the curve was smoothed by a fast Fourier transform (FFT), and the baseline level was adjusted. Then, we obtained smooth Raman spectral data with a high signal-to-noise ratio (Figures 5 and 6).

### 2.5. Statistical Analysis

Independent-samples *t*-tests were used to compare the integrated energy ratio and peak intensity ratio between the EGC and nonlesional tissue samples using SPSS 19 software. Then, the receiver operating characteristic (ROC) curve was drawn, and the area under the curve (AUC) was calculated.

## 3. Results

The average Raman spectra of the lesional and surrounding nonlesional tissues in EGC ESD specimens are shown in Figure 5. Significant Raman peaks corresponding to the nonlesional tissues were observed at 826 cm^−1^ (tyrosine), 852 cm^−1^ (proline, tyrosine), 891 cm^−1^ (saccharide), 992 cm^−1^ (unknown), 1103 cm^−1^ (phenylalanine), 1171 cm^−1^ (phenylalanine, tyrosine), 1206 cm^−1^ (tyrosine), 1234 cm^−1^ (amide III), 1292 cm^−1^ (cytosine), 1417 cm^−1^ (C=C stretching in quinoid ring), 1479 cm^−1^ (amide II), 1517 cm^−1^ (*β*-carotene accumulation), 1560 cm^−1^ (tryptophan), 1634 cm^−1^ (amide I), 1678 cm^−1^ (NADH), and 1729 cm^−1^ (ester group). In contrast, significant Raman peaks corresponding to the lesional tissues, including HGIN and adenocarcinoma, were observed in the Raman spectra at 826 cm^−1^, 888 cm^−1^, 977 cm^−1^, 987 cm^−1^, 1101 cm^−1^, 1171 cm^−1^, 1209 cm^−1^, 1236 cm^−1^, 1294 cm^−1^, 1324 cm^−1^ (collagen and purine bases of DNA), 1413 cm^−1^, 1517 cm^−1^, 1562 cm^−1^, and 1682 cm^−1^.  Figure 6 shows the average Raman spectra of the HGIN and adenocarcinoma tissues. As shown in Figure 6, the Raman spectrum of the HGIN tissues exhibits significant peaks at 826 cm^−1^, 838 cm^−1^ (amine deformation vibrations), 849 cm^−1^, 861 cm^−1^, 875 cm^−1^ (tryptophan), 889 cm^−1^, 987 cm^−1^, 1101 cm^−1^, 1171 cm^−1^, 1209 cm^− 1^, 1235 cm^−1^, 1253 cm^−1^ (C-O4 aromatic stretching), 1324 cm^−1^, 1413 cm^−1^, and 1562 cm^−1^, while the Raman spectrum of the adenocarcinoma tissues displays significant peaks at 820 cm^−1^ (structural protein modes of tumors), 849 cm^−1^, 861 cm^−1^, 875 cm^−1^, 887 cm^−1^, 948 cm^−1^ (single-bond stretching vibrations of the amino acids proline and valine and polysaccharides), 977 cm^−1^, 1102 cm^−1^, 1171 cm^−1^, 1209 cm^−1^, 1236 cm^−1^, 1293 cm^−1^, 1323 cm^−1^, 1413 cm^−1^, 1517 cm^−1^, 1531 cm^−1^ (carotenoid, absent from the normal tissue spectrum), and 1562 cm^−1^. The assignments for the peaks are given in Table 1 [14–20].

(1) There were obvious differences between the average Raman spectra of the lesional and nonlesional tissues in the EGC ESD specimens, as shown in Figure 5. The average Raman spectrum of the lesional tissues fluctuated less than that of the nonlesional tissues. In the range of 800-1350 cm^−1^, the peak intensity of the nonlesional tissues was obviously higher than that of the lesional tissues, while the peak intensity in the range of 1350-1800 cm^−1^ displayed the opposite trend. Compared with those of the nonlesional tissues, the Raman peaks of the lesional tissues at 826 cm^−1^, 1171 cm^−1^, and 1517 cm^−1^ remained fixed. In contrast, the Raman shifts of the spectral peaks of the lesional tissues at 888 cm^−1^, 1101 cm^−1^, and 1413 cm^−1^ moved toward lower wavenumbers than those of the nonlesional tissues at 891 cm^−1^ (saccharide), 1103 cm^−1^ (phenylalanine), and 1417 cm^−1^ (C=C stretching in quinoid ring), while the Raman shifts of the spectral peaks of the lesional tissues at 1209 cm^−1^, 1236 cm^−1^, 1294 cm^−1^, 1480 cm^−1^, 1562 cm^−1^, and 1682 cm^−1^ moved toward higher wavenumbers than those of the nonlesional tissues at 1206 cm^−1^ (tyrosine), 1234 cm^−1^ (amide III), 1292 cm^−1^ (cytosine), 1479 cm^−1^ (amide II), 1560 cm^−1^ (tryptophan), and 1678 cm^−1^ (NADH). Additionally, a characteristic peak was observed at 1324 cm^−1^ in the average Raman spectrum of the lesional tissues, which can be assigned to the purine bases in DNA.

The average Raman spectrum of the HGIN tissues was very close to that of the adenocarcinoma tissues in the lesional area of the EGC ESD specimens, as shown in Figure 6. The integral shape and peak fluctuation were very similar. Among the peaks observed in the spectra, the Raman shifts of the spectral peaks of the HGIN and adenocarcinoma tissues at 849 cm^−1^, 861 cm^−1^ (tyrosine), 875 cm^−1^ (tryptophan), 1171 cm^−1^, 1209 cm^−1^, 1413 cm^−1^, and 1562 cm^−1^ were the same. Furthermore, compared with those of the adenocarcinoma tissues at 887 cm^−1^, 1102 cm^−1^, 1236 cm^−1^, and 1323 cm^−1^, the Raman shifts of the spectral peaks of the HGIN tissues at 889 cm^−1^, 1101 cm^−1^, 1235 cm^−1^, and 1324 cm^−1^ were only slightly different. In the average Raman spectrum of the HGIN tissues, peaks were clearly observed at 826 cm^−1^, 838 cm^−1^ (amine deformation vibrations), and 1253 cm^−1^ (C-O4 aromatic stretching), while no related peaks were observed in the adenocarcinoma tissue spectrum. Additionally, peaks were observed at 820 cm^−1^ (structural protein modes of tumors), 948 cm^−1^ (single-bond stretching vibrations for the amino acids proline and valine and polysaccharides), 1293 cm^−1^, 1517 cm^−1^, and 1531 cm^−1^ (carotenoid, absent from the normal tissue spectrum) in the adenocarcinoma tissues, while no corresponding peaks were observed at 820 cm^−1^, 948 cm^−1^, and 1293 cm^−1^ in the HGIN tissues, and the related peaks at 1517 cm^−1^ and 1531 cm^−1^ were not obvious.

(2) The Raman spectra of the lesional and nonlesional tissues were analyzed by the integral energy ratio. The noncontinuous frequency band (E1500-1600 cm^−1^/E1050-1150 cm^−1^) and the continuous frequency band (E1350-1500 cm^−1^/E1200-1350 cm^−1^) were selected. The two sets of integral energy ratio data acquired from the lesional and nonlesional tissues were analyzed by independent-samples *t*-test, revealing a significant difference between the two sets of data (*P* < 0.01). The ROC curve was drawn by the SPSS 19 software. The AUC of the integral energy ratio in the noncontinuous frequency band (E1500-1600 cm^−1^/E1050-1150 cm^−1^) was 0.992, meaning that with this noncontinuous frequency band, the accuracy of identifying EGC was 99.2%, and the corresponding sensitivity and specificity were 93.9% and 95.5%, respectively. The AUC of the integral energy ratio in the continuous frequency band (E1350-1500 cm^−1^/E1200-1350 cm^−1^) was 0.996, meaning that with this continuous frequency band, the accuracy of identifying EGC was 99.6%, and the corresponding sensitivity and specificity were 97.0% and 95.5%, respectively.

Then, the Raman spectra of the HGIN and adenocarcinoma tissues were also analyzed based on the integral energy ratio. The noncontinuous frequency band (E1500-1600 cm^−1^/E1050-1150 cm^−1^) and the continuous frequency band (E1350-1500 cm^−1^/E1200-1350 cm^−1^) were selected. The two sets of integral energy ratio data acquired from the HGIN and adenocarcinoma tissues were also analyzed by independent-samples *t*-test, revealing no significant difference between the two sets of data (*P* > 0.05).

(3) The Raman spectra of the lesional and nonlesional tissues were analyzed by the traditional peak intensity ratio, and the peak intensity ratio of I1560 cm^−1^/I1103 cm^−1^ was selected. The two sets of peak intensity ratio data acquired from the lesional and nonlesional tissues were analyzed by independent-samples *t*-test, revealing a significant difference between the two sets of data (*P* < 0.01). The ROC curve was drawn by SPSS 19 software. The AUC of the peak intensity ratio of I1560 cm^−1^/I1103 cm^−1^ was 0.988, meaning that with this peak intensity ratio, the accuracy of identifying EGC was 98.8%, and the corresponding sensitivity and specificity were 93.9% and 91.9%, respectively.

Then, the Raman spectra of the HGIN and adenocarcinoma tissues were also analyzed by the traditional peak intensity ratio, and the peak intensity ratio of I1560 cm^−1^/I1103 cm^−1^ was selected. The two sets of peak intensity ratio data acquired from the HGIN and adenocarcinoma tissues were also analyzed by independent-samples *t*-test, revealing no significant difference between the two sets of data (*P* > 0.05).

**Figure 7:**
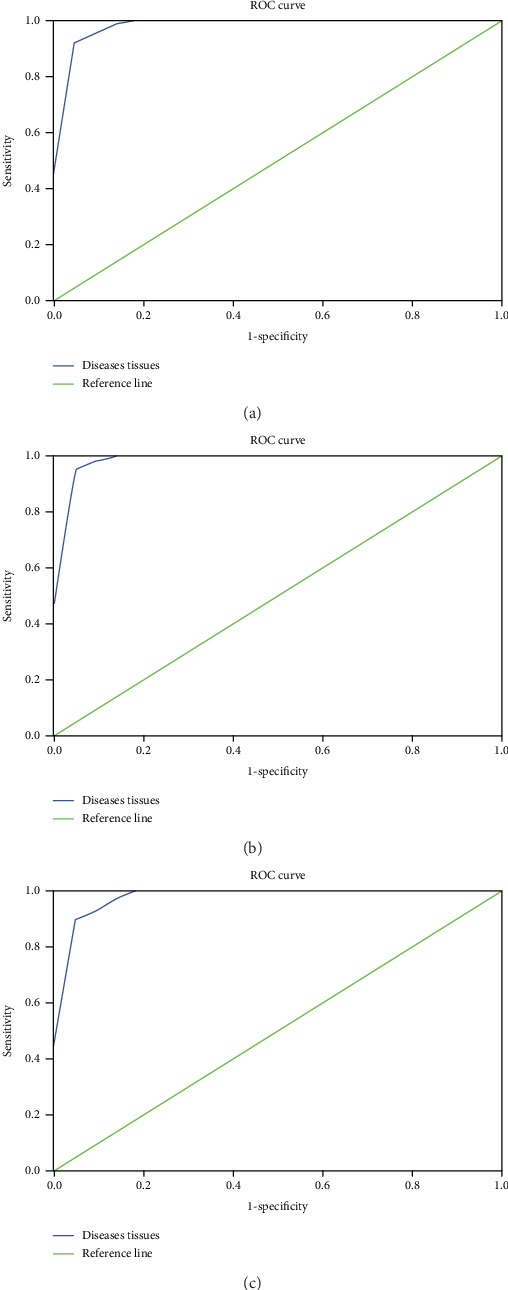
The ROC curves of (a) the integral energy ratio in the noncontinuous frequency band, (b) the integral energy ratio in the continuous frequency band, and (c) the peak intensity ratio of I1560 cm^−1^/I1103 cm^−1^.

**Figure 8:**
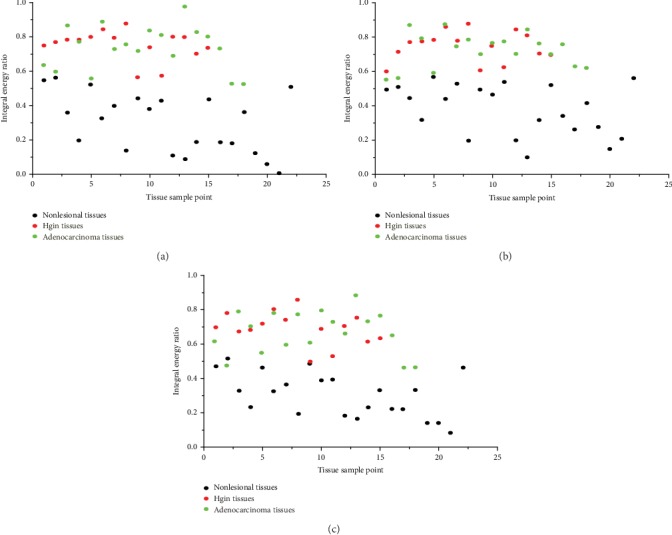
Scatter diagrams of (a) the integral energy ratio in the noncontinuous frequency band, (b) the integral energy ratio in the continuous frequency band, and (c) the peak intensity ratio.

The integral energy ratio and peak intensity ratio data are shown in Table 2. The ROC curves are shown in Figure 7. Scatter diagrams of the integral energy ratio and peak intensity ratio data are shown in Figure 8.

## 4. Discussion

The Raman effect was first discovered by the Indian scientist CV Raman and published in Nature on March 31, 1928 [21]. The Raman spectra of different molecules are different, which means that each molecule has its own fingerprint [22, 23]. Therefore, Raman spectroscopy is regarded as an emerging medical method for distinguishing cancerous tissues from normal tissues.

Studies on patients with gastric cancer or esophageal cancer have shown that the levels of phenylalanine in the serum, urine, and stomach contents of patients with tumors are higher than those in healthy people [24–28]. The reason may be related to significant changes in phenylalanine in the metabolic pathway, which leads to an increase in phenylacetylglutamine (a phenylalanine metabolite) [29]. A related study found that the level of phenylalanine in gastric cancer tissues showed an increasing trend, but there was no difference on statistical analysis [30], and another study confirmed that the level of phenylalanine in the tissues of patients with esophageal cancer was not significantly higher than that in the tissues of healthy people [31]. Other studies found that the tryptophan level in the tissues of patients with gastric cancer was significantly higher than that in normal tissues [32], while the tryptophan level in serum was decreased in gastric cancer tissue compared with normal tissue [25, 33–35], which may be related to the increased demand for and overutilization of amino acids in tumor tissues [28]. In this experiment, the peak intensity ratio of I1560 cm^−1^/I1103 cm^−1^ was significantly higher in the EGC tissues than in the nonlesional tissues, as demonstrated by independent-samples *t*-test. The average value in the nonlesional tissue group was 0.30, which is lower than that in the EGC tissue group (0.68). The *P* value was 0.000 < 0.01, showing a significant difference, and the accuracy, sensitivity, and specificity indicated in the ROC curve were 98.8%, 93.9%, and 91.9%, respectively.

The peak at 1560 cm^−1^ was assigned to tryptophan, and the peak at 1103 cm^−1^ was assigned to phenylalanine, indicating that the tryptophan level in EGC tissues was greater than that in nonlesional tissues. Accordingly, tryptophan may serve as a potential tumor marker, providing a basis for the diagnosis of EGC, but further study is still necessary.

The Raman peaks of the lesional tissues at 891 cm^−1^ (saccharide), 1103 cm^−1^ (phenylalanine), and 1417 cm^−1^ (C=C stretching in quinoid ring) moved to lower wavenumbers than those of nonlesional tissues, while the other peaks at 1206 cm^−1^ (tyrosine), 1234 cm^−1^ (amide III), 1294 cm^−1^ (cytosine), 1479 cm^−1^ (amide II), 1560 cm^−1^ (tryptophan), and 1678 cm^−1^ (NADH) moved to higher wavenumbers. This upward shift indicates an increase in the energy of vibration. It is believed that changes in the structure of tumor tissue may occur and that proteins, nucleic acids, and other compounds may become more stable because of the introduction of a new ligand or the activation of a surrounding ligand. This result confirms that compared with nonlesional tissues, tumor tissues show obvious changes in terms of the structure of proteins, saccharides, and nucleic acids. A characteristic peak was observed at 1324 cm^−1^ in the lesional tissues, which was assigned to collagen and the purine bases in DNA, but it was not observed in the nonlesional tissues, indicating that it may be related to changes in the DNA structure in tumor tissues and active nuclear division.

A significant peak was observed at 992 cm^−1^ in the nonlesional tissues. A peak could also be observed at 987 cm^−1^ in the HGIN tissues and at 977 cm^−1^ in the adenocarcinoma tissues, showing a trend toward lower wavenumbers. There have been no relevant reports in the literature regarding the substance to which the characteristic peak may be assigned. The other characteristic peaks were clearly characterized, and there was good correspondence between the peaks of the lesional and nonlesional tissues. Therefore, it was assumed that there was one kind of molecule with a peak located near 992 cm^−1^ and that the peak shifted from 992 cm^−1^ in the lesional tissues because of the effects of HGIN and adenocarcinoma. However, this assumption has not yet been confirmed. Another assumption was related to the use of ESD biopsy specimens in this experiment, while most studies reported to date have used biopsy specimens obtained by endoscopy. The histopathological results clearly indicated negative peripheral margins, meaning that the surrounding tissue was nonlesional tissue; however, the surrounding tissue may have been affected by the diseased tissue in the center, resulting in atrophy or intestinalization and causing certain changes at the molecular level because of the proximity to the diseased tissue, which may have caused the appearance of the characteristic peak at 992 cm^−1^. The above two assumptions still need to be confirmed by further experiments, and the next research step could be exploring differences in Raman spectra between normal and nonlesional tissues.

In this experiment, the integral energy ratio and the peak intensity ratio were used as indicators to diagnose EGC. The results show that the accuracy of the peak intensity ratio of I1560 cm-1/I1103 cm^−1^ was high, but the sensitivity and specificity were lower than those of the integral capacity ratio. Additionally, the accuracy, sensitivity, and specificity of the integral energy ratio in the noncontinuous frequency band and continuous frequency band were all better than those of the traditional mode peak intensity ratio (noncontinuous frequency band: accuracy, 99.2%; sensitivity, 93.9%; and specificity, 95.5%; continuous frequency band: accuracy, 99.6%; sensitivity, 97.0%; and specificity, 95.5%). Moreover, using the peak intensity ratio of a single peak increases the occurrence of errors, while calculating the integral energy ratio within a certain area reduces the occurrence of errors. Therefore, the integral energy ratio of the Raman spectrum could be considered an effective indicator for the diagnosis of EGC.

Currently, the combination of ME-NBI and traditional white-light endoscopy is usually used to diagnose EGC in the clinic. When suspicious lesions are found under white-light endoscopy, observation under ME-NBI is often recommended in addition to biopsy. A meta-analysis in 2015 [36], including 2171 patients from 14 studies, indicated that the pooled sensitivity and specificity for ME-NBI in the diagnosis of EGC were 86% and 96%, respectively, with an AUC of 0.9623. The sensitivity and specificity of traditional white-light endoscopy reported in this study were only 57% and 79%, respectively. Another meta-analysis in 2018 [37], including 5398 patients from 9 studies, also showed that the sensitivity and specificity of ME-NBI in distinguishing between cancerous and noncancerous gastric lesions were 88% and 96%, respectively, with an AUC of 0.97, indicating the accuracy of ME-NBI in distinguishing between cancerous and noncancerous gastric lesions. Compared with the traditional white-light endoscopy, the sensitivity and specificity of the fiber optic Raman system and ME-NBI are significantly higher. While the latter two methods have similar specificity, the accuracy and sensitivity of the fiber optic Raman system are obviously higher than those of ME-NBI, which proves that the fiber optic Raman system represents a high-accuracy diagnostic method.

The integral shape and peak distribution of the Raman spectra of the HGIN and adenocarcinoma tissues were very similar, and approximately 50% percent of all peaks were completely consistent. Among the other peaks, some showed only slight changes (1-2 cm^−1^) in their Raman shift, and certain peaks were different from their distribution, which proves that there are certain differences between HGIN and adenocarcinoma tissues on Raman spectroscopy. The peak intensities in the two sets of data were also similar. However, the integral energy ratio and traditional peak intensity ratio were also used to analyze the differences between the HGIN and adenocarcinoma tissues, and no significant differences between the two groups were found, which means that there were no significant differences between the Raman spectra of the two pathological types. Considering that a small sample size may not fully reflect the differences in the Raman spectra between the two groups, expanding the sample size is required to further explore the differences between the two groups.

This study explored only the differences between HGIN and adenocarcinoma tissues. Answering the questions of whether the Raman spectra of EGC tissues of different pathological types are different and whether the Raman spectra of EGC and advanced gastric cancer tissues are different requires further study. This study preliminarily proves that it is feasible to identify the differences between HGIN and adenocarcinoma tissues and peripheral nonlesional tissues in ESD specimens by Raman spectroscopy, which provides a research basis for defining lesion boundaries by Raman spectroscopy. However, large amounts of sample data still need to be collected for analysis. In the next research step, we will collect more sample data, reduce the error, combine in vivo detection with in vitro detection, establish an initial diagnostic database of Raman spectra, and establish a standard for the diagnosis of EGC by Raman spectroscopy.

## Figures and Tables

**Table 1:** Raman peak assignments.

Raman shift				
Nonlesional tissues	HGIN tissues	Adenocarcinoma tissues	Lesional tissues	Assignment
		820 cm^−1^		Structural protein modes of tumors
826 cm^−1^	826 cm^−1^		826 cm^−1^	Tyrosine
	838 cm^−1^			Amine deformation vibrations
	849 cm^−1^	849 cm^−1^		
852 cm^−1^				Proline, tyrosine
	861 cm^−1^	861 cm^−1^		Tyrosine, collagen (859 cm^−1^)
	875 cm^−1^	875 cm^−1^		Tryptophan
		887 cm^−1^		
			888 cm^−1^	
	889 cm^−1^			
891 cm^−1^				Saccharide
		948 cm^−1^		Single-bond stretching vibrations for the amino acids proline and valine and polysaccharides
	1101 cm^−1^		1101 cm^−1^	
		1102 cm^−1^		
1103 cm^−1^				Phenylalanine
1171 cm^−1^	1171 cm^−1^	1171 cm^−1^	1171 cm^−1^	Phenylalanine, tyrosine
1206 cm^−1^				Tyrosine
	1209 cm^−1^	1209 cm^−1^	1209 cm^−1^	
1234 cm^−1^				Amide III
	1235 cm^−1^			
		1236 cm^−1^	1236 cm^−1^	
	1253 cm^−1^			C-O4 aromatic stretching
1292 cm^−1^				Cytosine
		1293 cm^−1^		
			1294 cm^−1^	
		1323 cm^−1^		
	1324 cm^−1^		1324 cm^−1^	Collagen and purine bases of DNA
	1413 cm^−1^	1413 cm^−1^	1413 cm^−1^	
1417 cm^−1^				C=C stretching in quinoid ring
1479 cm^−1^				Amide II
1517 cm^−1^		1517 cm^−1^	1517 cm^−1^	*β*-carotene accumulation
		1531 cm^−1^		Carotenoid, absent from normal tissues
1560 cm^−1^				Tryptophan
	1562 cm^−1^	1562 cm^−1^	1562 cm^−1^	
1634 cm^−1^				Amide I
1678 cm^−1^				NADH
			1678 cm^−1^	
1729 cm^−1^				Ester group

**Table 2:** Integral energy ratio and peak intensity ratio values.

E1/E2				E3/E4				I1560 cm^−1^/I1103 cm^−1^			
Can1	Can2	Nor		Can1	Can2	Nor		Can1	Can2	Nor	
0.63	0.75	0.55	0.12	0.55	0.60	0.49	0.27	0.61	0.70	0.47	0.14
0.60	0.77	0.56	0.06	0.56	0.72	0.51	0.15	0.48	0.78	0.51	0.14
0.87	0.78	0.36	0.01	0.87	0.77	0.44	0.21	0.79	0.67	0.33	0.08
0.77	0.78	0.20	0.51	0.79	0.78	0.32	0.56	0.71	0.68	0.24	0.46
0.56	0.80	0.53		0.59	0.78	0.57		0.55	0.72	0.46	
0.89	0.85	0.33		0.87	0.86	0.43		0.78	0.81	0.33	
0.73	0.79	0.40		0.74	0.78	0.53		0.60	0.74	0.36	
0.75	0.88	0.14		0.79	0.88	0.19		0.77	0.86	0.19	
0.71	0.56	0.44		0.70	0.61	0.49		0.61	0.50	0.48	
0.84	0.74	0.38		0.77	0.75	0.46		0.80	0.69	0.39	
0.81	0.57	0.43		0.77	0.62	0.54		0.73	0.53	0.39	
0.69	0.80	0.11		0.70	0.84	0.19		0.66	0.71	0.18	
0.98	0.80	0.09		0.84	0.81	0.09		0.88	0.75	0.17	
0.83	0.70	0.19		0.76	0.70	0.32		0.73	0.62	0.23	
0.80	0.74	0.43		0.70	0.70	0.52		0.77	0.63	0.33	
0.73		0.19		0.76		0.34		0.65	0.70	0.22	
0.53		0.18		0.63		0.26		0.46	0.78	0.22	
0.52		0.36		0.62		0.41		0.47	0.67	0.33	

E1: E1500-1600 cm^−1^; E2: E1050-1150 cm^−1^; E3: E1350-1500 cm^−1^; E4: E1200-1350 cm^−1^; Can1: Adenocarcinoma tissues; Can2: High-grade intraepithelial neoplasia tissues.

## Data Availability

The data used to support the findings of this study are available from the corresponding author upon request.
